# HDACi-Loaded Nanoliposomes Enhance the Diversity of Antibody CDRs in Rabbits and the Application in Antibody Preparation

**DOI:** 10.1021/acsomega.5c07628

**Published:** 2026-04-22

**Authors:** Ling Chen, Li Huang, Xingbo Yan, Panpan Zhang, Enben Su, Yu Zhang

**Affiliations:** † State Key Laboratory of Digital Medical Engineering, Jiangsu Key Laboratory for Biomaterials and Devices, School of Biological Science and Medical Engineering & Basic Medicine Research and Innovation Center of Ministry of Education, Zhongda Hospital, 12579 Southeast University, Nanjing 211102, China; ‡ Getein Biotechnology Co., Ltd., Nanjing 211505, China

## Abstract

Although a variety of novel strategies have been used to select antibody-producing cells efficiently either from natural B cell collections or from plasma cell collections postimmunization, the production of monoclonal antibodies against weak antigens with high affinity and special epitopes for diagnostic or therapeutic applications remains fraught with challenges and uncertainty. In this report, we proposed an effective method for generating monoclonal antibodies from rabbits by using histone deacetylase inhibitor (HDACi) to improve antibody gene diversity, particularly the diversity of the complementarity-determining regions (CDRs) which determines the specificity of the antibody. HDACi was encapsulated in nanoliposomes to form an immunomodulator, which was administered to neonatal rabbits during the ontogeny of the immune systems. It was observed that the number of antigen-specific plasma cells in these treated rabbits was increased, and more importantly, the antibody gene diversity, especially the CDRs, was proven to have increased after 3 times of antigen immunization compared with the untreated group, probably due to the promoted efficiency of gene conversion. The results indicated that the histone modification mediated by HDACi treatment has an effect of promoting diversification of the antibody genes in treated animals. The nanoliposomes as a carrier of HDACi helped to increase the utilization rate of the loaded drugs, reduce the total dosage of administrations, and extend the time interval between administrations, making the drug feasible for newborn animals. This new method enables animals to generate antibodies against different epitopes of weakly immunogenic antigens, such as peptide antigens, and makes it possible to select antibodies with better performance for subsequent application in diagnostic reagents.

## Graphical abstract

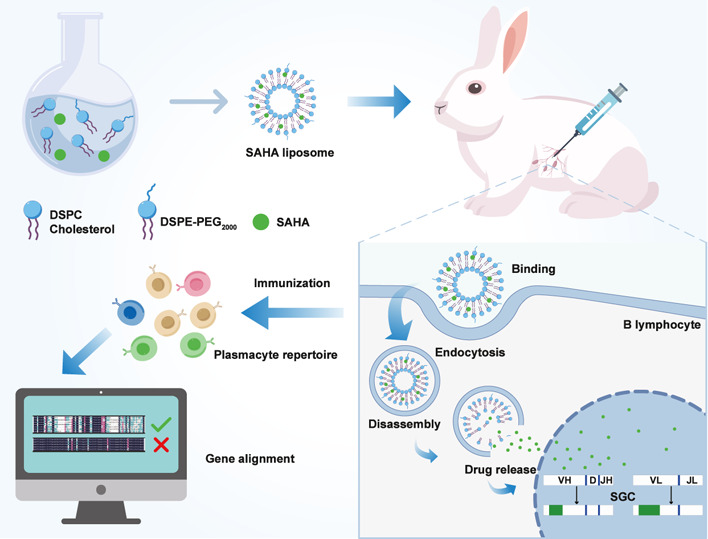

## Introduction

Antibodies have been widely used in scientific research, diagnosis, and therapy. In order to obtain antibodies with potential utility, many methods have been developed to prepare monoclonal antibodies, including hybridoma,^1^ phage display,^2–4^ single B-cell cloning,^5^ etc. The sources of monoclonal antibodies are not limited to mice but also humans, rabbits, alpacas, and many other species. However, these existing methodologies still have limitations in preparing antibodies with weakly immunogenic antigens such as peptide antigens.^6,7^ One of the facts we discovered is the limited diversity of the antibody library in immunized animals, which significantly hinders the acquisition of high-performance antibodies.

In many species, the diversity of antibodies generated by the immunoglobulin V­(D)­J gene rearrangement is limited. Therefore, post-rearrangement mechanisms, including gene conversion (GCV) and/or somatic hypermutation (SHM), take place in gut-associated lymphoid tissues (GALT) to further diversify the preimmune repertoire.^8^ Most of the somatic diversification in mammals is due to somatic mutation rather than gene conversion.^9^ Gene conversion appears as a special mechanism and is only used by a few species, mainly birds and rabbits.^8^ The diversity of rabbit mature B cells develops through a unique gene conversion mechanism,^10^ which occurs in the intestinal-associated lymphoid tissues from 2 days to 6 weeks after birth.^11,12^ These postnatal mechanisms enable rabbits to produce antibodies with high specificity and affinity for specific antigens. But it is still difficult to produce applicable antibodies against weak antigens, due to the lack of immunogenic epitopes recognizable by B-cell receptors on the host’s B cells.^13^ To solve this problem, we proposed further improving the diversity of rabbit antibody complementarity-determining regions (CDRs) by promoting gene conversion using special immunomodulators in order to develop effective antibodies more efficiently.

Two regulatory factors associated with the gene conversion (GCV) mechanism have been reported that can modulate the activity of this procedure. Natsuki et al. revealed that TET3 induced hypermethylation in some Ig pseudogene templates and exhibited a marked reduction in GCV activity in Ig-variable regions that resulted in less Ig diversification.^14^ However, Paulina et al. demonstrated that Bach2 promotes GCV by increasing the expression of activation-induced cytidine deaminase (AID).^15^

Acetylation of histone proteins has been associated with active transcription in the way of weakening histone–DNA interaction by neutralizing lysine’s positive charge to enhance chromatin accessibility. Histone acetylation is regulated by two opposing enzyme classes: histone acetyltransferases and histone deacetylases (HDACs).^16^ Based on this theory, histone deacetylase inhibitors (HDACi) were developed to enhance the transcription and expression of tumor suppressor genes and have been demonstrated to play a role in the anticancer gene activation in clinical treatment, and some types of chemical forms of HDACi have received regulatory approval and achieved encouraging clinical results.^17–21^ It also reported that some HDACi molecules could activate the antibody gene conversion of B cells in vitro, which was then used in the screening of antibodies for special antigens,^22^ but the affinity of the antibodies was not strong enough for applications in diagnostic reagents.

Here, suberoylanilide hydroxamic acid (SAHA), a kind of HDACi molecule, was applied to rabbits as an immunomodulator, and these rabbits were subsequently immunized with specific antigens to produce antibodies. To achieve efficient utilization and slow stable release of drugs in the body, we encapsulated SAHA in liposomes and performed intraperitoneal injection to make the drug act directly on lymphoid-associated tissues, where gene conversion occurs.^23,24^ Meanwhile, it was reported that liposomes as a carrier for drug delivery have a passive targeting effect on lymphoid tissues.^25^ Furthermore, the incorporation of PEG polymers on liposome surfaces can reduce the nonspecific adsorption of proteins and delay recognition and engulfment by the mononuclear phagocyte system, prolong the retention time of the drug in vivo, and thereby improve the bioavailability of the drug.^26^ Subsequently, the results of antibody genetic alignment in this research showed that the CDR gene diversity of antigen-specific antibodies obtained from the rabbits with immunomodulator treatment was significantly higher than that from the rabbits without treatment. Moreover, the nanoliposome-encapsulated formulation achieved the same immune effect as the free SAHA with a lower total dosage of the drug and fewer injections. This method expanded the scope of antibodies for the next screening steps. Thereby, the possibility of obtaining effective antibodies is increased from the source, especially for the weak antigens.

## Results

### HDACi Liposome Preparation and Characterization

We selected SAHA as the HDACi immunoregulator for the experiment. First, we prepared SAHA-encapsulated liposomes using DSPC, cholesterol, DSPE-PEG, and SAHA with the thin-film dispersion method, followed by ultrasonic crushing and homogenizer treatment. The average particle size of the obtained liposomes measured by dynamic light scattering (DLS) was 159 nm (PDI: 0.269).^26,27^ The zeta potential of the liposomes was −22.9 mV, and the liposomes presented an approximately spherical structure and had good dispersibility, as indicated by transmission electron microscopy (TEM) (Figure 1a–c). Accordingly, we prepared liposomes encapsulating fluorescein coumarin 6 (C6) to represent SAHA-LP for cell experiments in vitro with the same method. The particle size was 167 nm (PDI: 0.144), and zeta potential was −17.7 mV. The TEM image showed a similar appearance to SAHA-LP (Figure 1d–f). The entrapment efficiency (EE) was 76%, the loading content (LC) was 7.6%, and the cumulative release rate of SAHA was measured (Figure 1g). The stability of the liposomes in the buffer containing plasma proteins was determined. The liposomes were dissolved in phosphate-buffered saline (PBS) and PBS containing 10% fetal bovine serum (FBS), respectively, and their particle size fluctuated only within a small range within 72 h (Figure 1h). The anionic charge on the liposomes increases the repulsion forces between liposomes and prevents nonspecific binding of plasma proteins which are usually negatively charged, thereby preventing liposomal aggregation and ensuring the good stability and blood compatibility of the liposomes in vivo.^28^ This formulation of SAHA-LP provides the possibility of reducing the dosage and the frequency of injections in subsequent experiments in animals.

**Figure 1:**
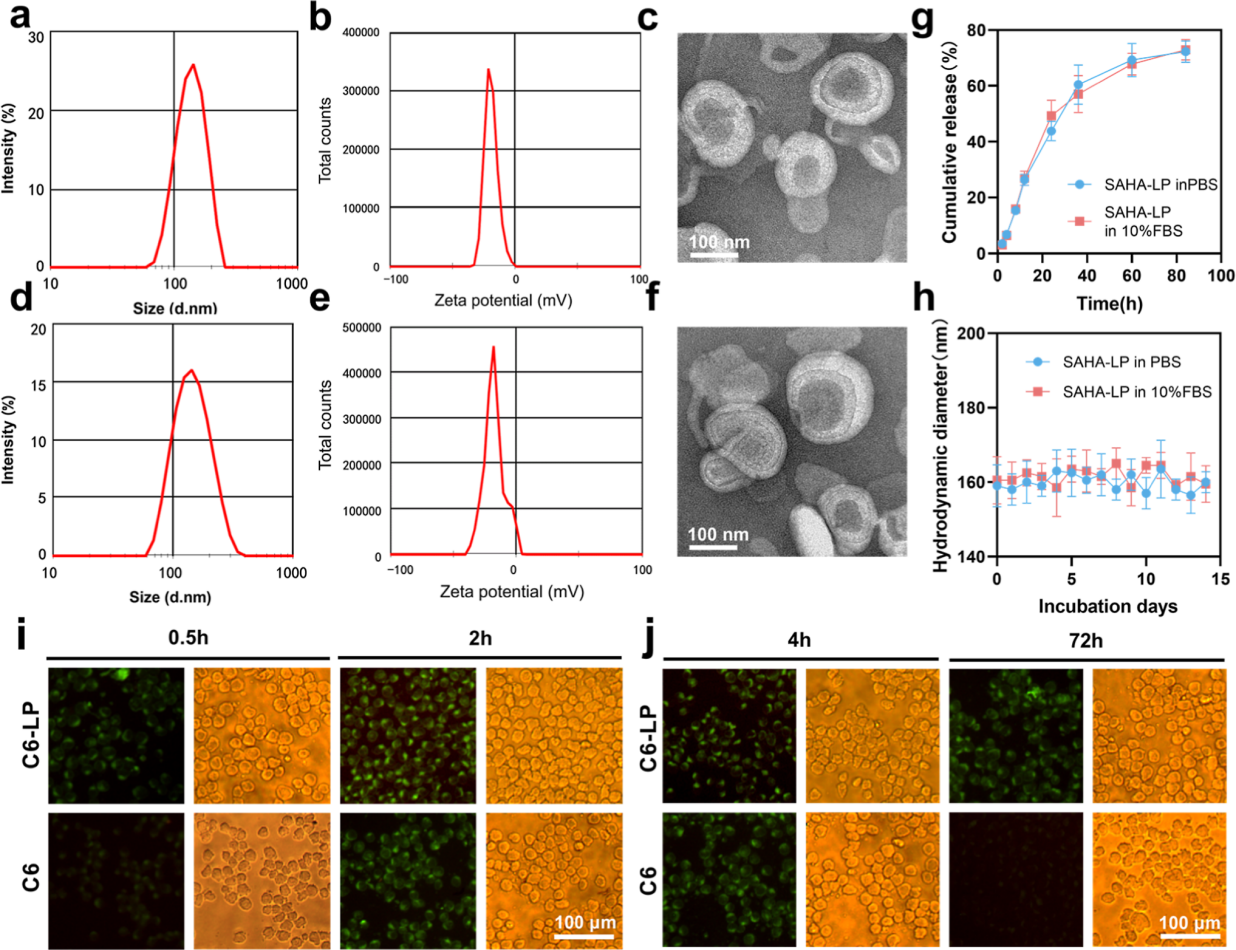
Characterization of SAHA-LP and C6-LP. (a,d) Particle size distribution, (b,e) zeta potential, and (c,f) TEM images of SAHA-LP (a–c) and C6-LP (d–f) (scale bar = 100 nm). (g) Release profiles of SAHA-LP and C6-LP in 84 h. (h) Changes in particle size of SAHA-LP in PBS and 10% FBS over 2 weeks. (i,j) Immunofluorescence images of C6-LP and C6 interaction and retention in cells by incubation with SP2/0 cells for different times (scale bar = 100 μm) (mean ± SD, *n* = 3 per group).

To investigate whether SAHA-LP can successfully enter cells and tissues in order to exert regulatory effects, we observed the process of C6-LP entering SP2/0 cells and then metabolizing it to represent the performance of SAHA-LP in vitro. At the same time, we observed its retention time in cells to determine the interval and dosage for in vivo use.

In vitro cell uptake assay showed that C6-LP can be taken up by cells in less than 0.5 h and retained within them for at least 72 h, significantly longer than the C6 group. Besides, the cell viability rate in the C6-LP group was >95%, and during the subsequent cultivation, they were able to proliferate normally just like those in the C6 group. It indicates that liposomes can be a good carrier for delivering immunomodulator drugs to cells.

### SAHA-Liposomes Assisted Immune Response

The drug-loaded liposomes SAHA-LP and free SAHA were administered to newborn rabbits of 1 week after birth in two experimental groups, respectively, according to the procedure shown below (Figure 2a, blue), while the rabbits in the control group were not treated (Figure 2a, red). Subsequently, the rabbits in experimental and control groups were all immunized with a 32-amino acid antigen of brain natriuretic peptide (BNP). After 3 immunizations, the rabbit serum titer was detected, and it showed that the rabbits in the two immunomodulator groups yielded higher serum titer compared to the control group, while both experimental groups were at a similar level (Figure 2e). As we expected, the method of encapsulating an equivalent dose of SAHA into liposomes to reduce injecting times (once every 3 days) can achieve the same effect compared with free drugs (once a day), indicating that liposomes lead to sustained release of the drug and higher bioavailability in the body.

**Figure 2:**
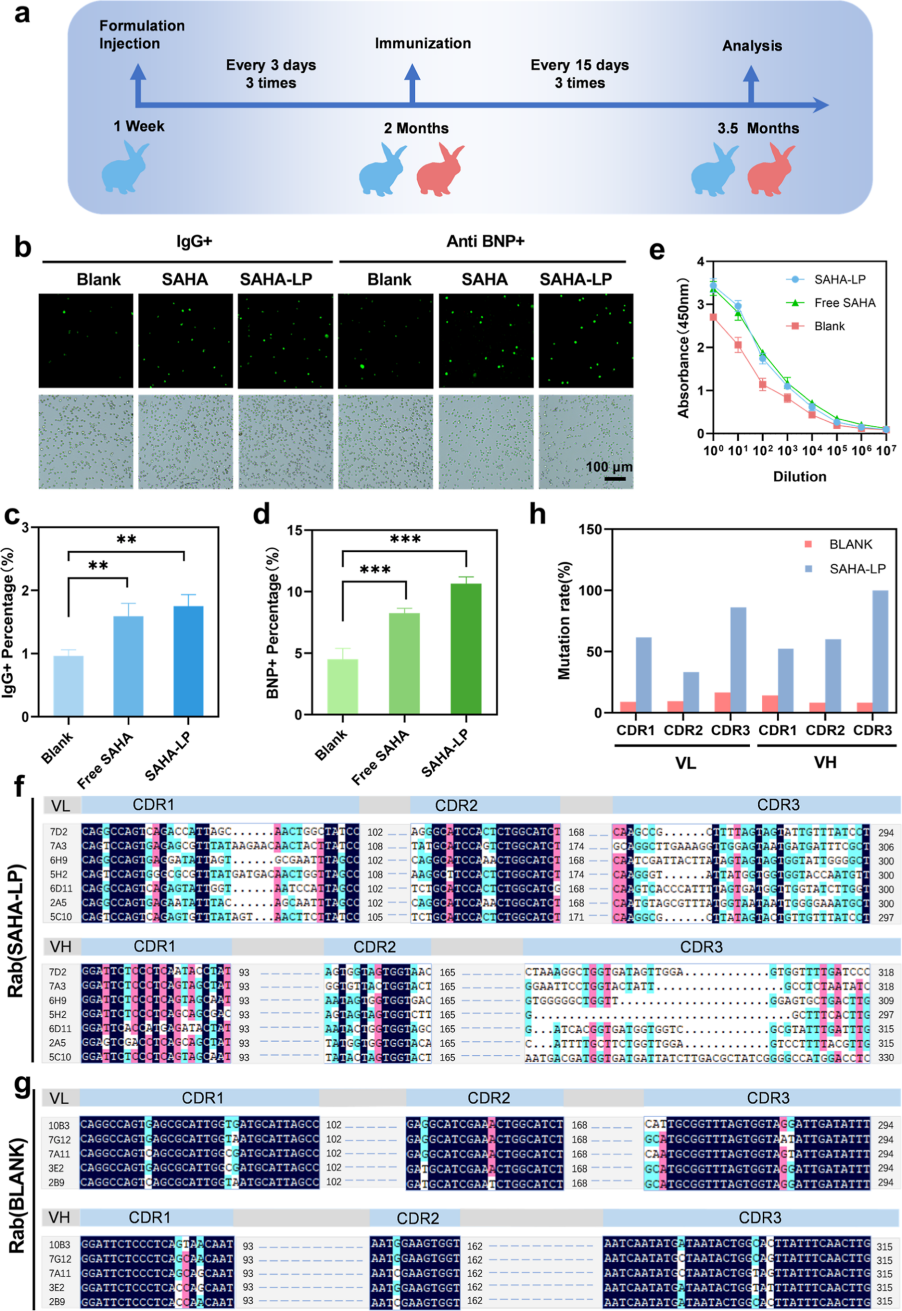
Identification of the effects of immunomodulators on rabbit immune response. (a) Schematic diagram of formulation injection and immunization of rabbits. (b) Immunofluorescence images of plasma cells from SAHA-LP-treated and untreated rabbits reacted with goat antirabbit IgG and BNP antigen (scale bar = 100 μm). (c) Proportion of IgG+ cells among spleen cells by immunofluorescence assay. (d) Proportion of BNP antibody-secreting cells among IgG+ cells by immunofluorescence assay. (e) Serum titer of rabbits injected with SAHA-LP and free SAHA by indirect ELISA. (f,g) Multiple alignments of antibody genes of heavy- and light-chain CDRs obtained from rabbits in the experimental group (f) and control group (g) using DNAMAN software. (h) Mutation rate of the CDRs in the variable areas of heavy and light chains of antibody genes (mean ± SD, *n* = 3 per group; **P < 0.01 and ***P < 0.001).

The immunofluorescence assays showed that the proportion of total IgG+ plasma cells and the proportion of BNP antibody-secreting cells among IgG+ plasma cells were both higher in the free SAHA and SAHA-LP treatment groups than those in the untreated group (Figure 2b–d). These results indicated that SAHA-LP treatment can promote the immune response of the B cell immune system, which, therefore, can produce more functional plasma cells and antibodies against the BNP antigen.

### Antibody Screen and Gene Sequencing

We collected spleen cells from the immunized rabbits, which subsequently fused with mouse myeloma cells to form heterologous hybridomas. Then, the antigen-reactive hybridomas were selected and subcloned to single clones.

After antigen detection on the supernatant of monoclonal cells, we selected 7 highly positive clones in the SAHA-LP-treated group and 5 in the untreated group for antibody gene amplification and sequencing. Here, we optimized the method for cellular RT-PCR and screened a reagent kit that can perform amplification in the case of a small number of cells.

We sequenced the RT-PCR products of the antibody variable region genes of the positive cells. According to the gene sequencing results, we compared the gene alignment data of the experimental group with the control group (Figure 2f–h). The genes from the SAHA-LP group accumulated more mutations in complementary determining regions (CDRs) 1–3 (62 mutations/96 nt in L-chain CDRs and 68 mutations/84 nt in H-chain CDRs) than those in the control group (10 mutations/84 nt in L-chain CDRs and 7 mutations/69 nt in H-chain CDRs). Moreover, it was varied to the length of the CDR1 and CDR3 sequences in the SAHA-LP group which were not found in the control group. These mutations in the CDR regions resulted in different amino acid sequences and expanded the total antibody repertoire.

### Antibody Expression and Characterization

In order to obtain antibodies constantly, we cloned each variable region of genes of selected antibodies into the expression vectors and transfected HEK 293 cells to prepare antibodies.

We detected the titers and affinities of the 7 rabbit antibodies with the strongest reaction to the antigen in the SAHA-LP group and 5 in the control group. The ELISA titers of the antibodies in SAHA-LP group are generally higher than the mouse antibodies prepared previously and even the rabbit antibodies from the control group (Figure 3a). Moreover, the affinities of the obtained rabbit antibodies were detected and showed that the *K*_D_(*K*_d_/*K*_a_) value of the antibodies can reach 10^–12^ mol/L, while the affinities of mouse antibodies are generally in the range 10^–9^ to 10^–10^ mol/L (Figure 3b). These results demonstrated the feasibility that rabbits can produce antibodies with high affinity against peptide antigens and that SAHA-LP helps to select useful antibodies from a more diverse antibody library.

**Figure 3:**
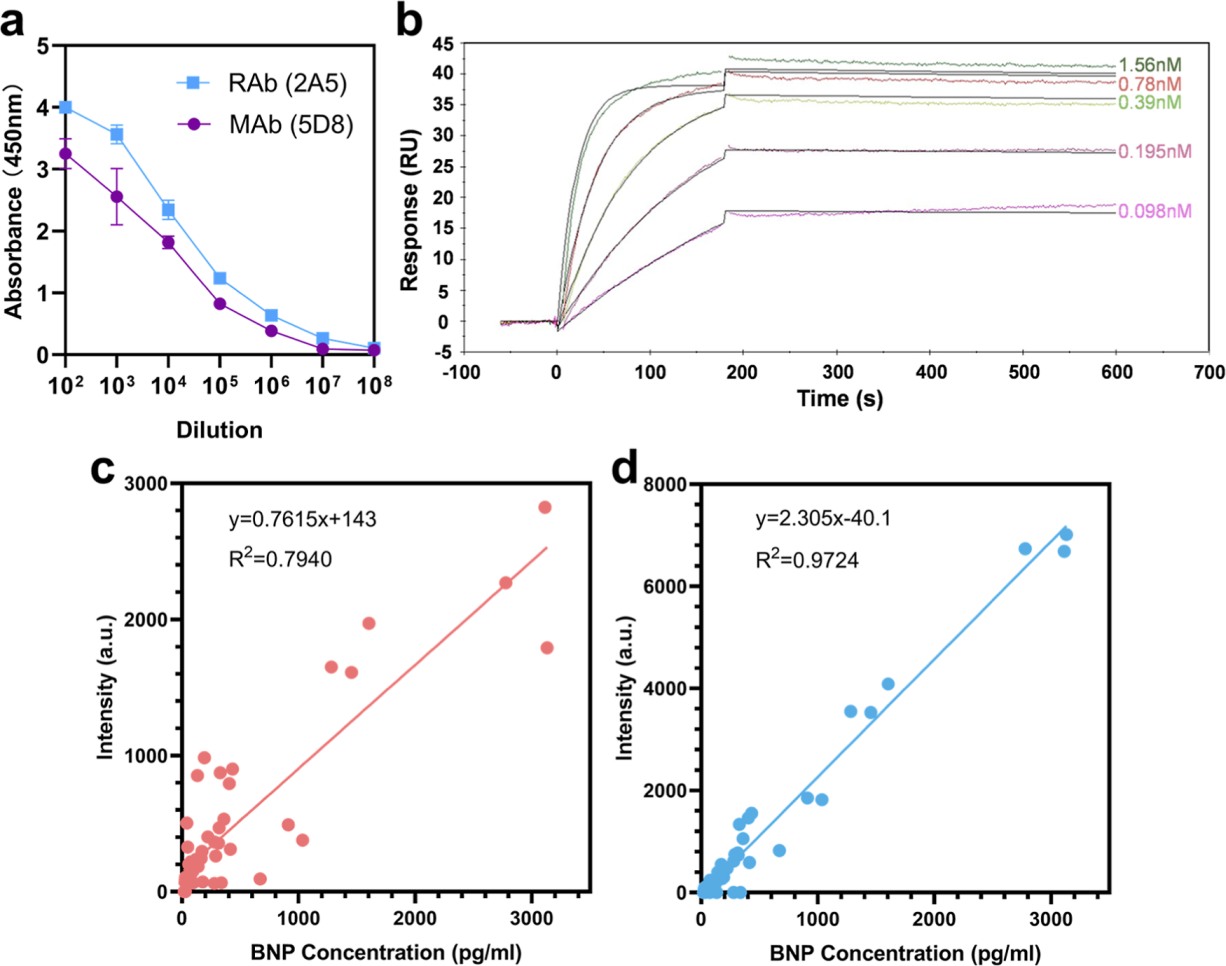
Characterization and application of selected antibodies. (a) ELISA titers of rabbit antibody 2A5 and mouse antibody 5D8 which were used in FICA. (b) KD measurement of Rab against BNP by surface plasmon resonance. The dilutions of Rab from 1.56 nM to 0.098 nM were tested against 100 nM of BNP at room temperature. (c,d) Calibration curves for simultaneous multiplexed detection of BNP by FICA using two mouse antibodies (c) and rabbit–mouse antibodies (d) (mean ± SD, *n* = 3 per group).

### Establish Fluorescence Immunochromatographic Assays with Prepared Antibodies

These rabbit antibodies were paired separately with the previously obtained monoclonal antibodies from mice in the double-antibody sandwich ELISA. Rabbit antibody 2A5 and mouse antibody 5D8 were selected as the best performing pair.

Then, the two selected antibodies were used to set up a fluorescence immunochromatographic assay (FICA).^29,30^ Compared with the reagent prepared with two mouse antibodies, the new reagent using the mouse–rabbit antibodies is better in terms of sensitivity and consistency and has a wider detection range (Figure 3c,d). Moreover, the use of antibodies from different species avoided the interference of HAMA (human antimouse antibodies) present in the serum samples, thus providing higher accuracy.

## Conclusion and Discussion

Rabbits are considered a better choice than mice for antibody preparation owing to their greater genetic differences from humans, which can stimulate a stronger immune response against human antigens. However, the problem of preparing antibodies against weak antigens that lack strong immunogenic epitopes still needs addressing.

It is known that in the ontogeny of the rabbit immune cells, the early V­(D)­J recombination uses only a very small portion of genes, particularly in heavy chain; only two VH genes out of 200 for the heavy-chain locus, embedded in the VH cluster 20 kb upstream, are used in approximately 80% of B cells. Gene conversion as a post-rearrangement diversification mechanism plays a key role in expanding antibody gene repertoire after birth.^8^

In this article, we developed an immunomodulatory agent, SAHA-LP, for the treatment of 1 week old rabbits during the B cell gene conversion period. SAHA can effectively increase the CDR postnatal diversification of antibodies against the peptide antigen. Although the mechanism of this process is not investigated in this research, it is known that HDACi can lead to gene exposure, which promotes transcriptional activity.^31,32^ Budzynska et al. have demonstrated that AID plays a role in mediating gene conversion in rabbit antibody repertoire ontogeny.^15^ Does HDACi promote the transcription and expression of AID gene, thereby enhancing gene conversion? We will conduct more experiments to verify the assumptions about the mechanism.

Liposomes have been proven to be excellent drug carriers, and many liposomal formulations of targeted drugs are widely used in clinical or preclinical studies.^33–38^ In this study, nanoliposomes improved the cell uptake efficiency and intracellular retention duration of SAHA, ensuring the drug maintained an effective concentration over an extended period (approximately 3 days in our research), thereby enhancing drug utilization, reducing injection frequency, and minimizing the resulting irritation to newborn rabbits.

Additionally, due to the larger interstitial gaps in lymphatic endothelium compared to vascular endothelium, liposomes are selectively transported into lymphatic tissues by intraperitoneal administration, which may facilitate the prolonged exposure of immune cells to SAHA, consistent with the prior reports of nanoparticle lymphatic transport.^39,40^ This is supposed to be another reason that SAHA-LP could lead to a reduction in the total dosage of SAHA used.

Rabbits utilize gene conversion and SHM to diversify rearranged heavy-chain VH, DH, and light-chain VJ genes both in the appendix and other GALT.^41^ Here, we administered SAHA-LP via intraperitoneal injection so as to make the drug-loaded liposomes predominantly distributed in GALT and internalize into B cells directly, avoiding the whole-body distribution by intravenous injection.^24^ The measures mentioned above may have jointly contributed to the increase in the utilization rate of drugs, but we did not directly evaluate in vivo biodistribution or lymphatic targeting in this study.

This study has several limitations that warrant further in-depth research and exploration in our future work. First, antibody sequence diversity was evaluated from a limited number of antigen-reactive clones rather than by high-throughput repertoire sequencing and therefore does not represent a comprehensive repertoire-wide analysis. Second, the molecular mechanisms underlying increased CDR variability were not directly investigated; we did not measure AID expression, chromatin accessibility, or gene conversion events and thus cannot distinguish between enhanced gene conversion and altered SHM. Third, the in vivo biodistribution and lymphatic targeting of SAHA-loaded liposomes were not assessed. Finally, liposome encapsulation was used primarily to improve the dosing practicality and sustained exposure rather than to enhance the intrinsic biological activity of SAHA.

Briefly, this immunomodulatory agent establishes a new method for preparing antibodies targeting more epitopes of antigens, particularly providing a solution for obtaining antibodies against weak antigens such as peptide antigens. This method may also provide an alternative solution for the preparation of antibodies against small molecular antigens <1000 Da, which needs to be verified by more experiments.

## Experimental Section

### Materials

SAHA, C6 was purchased from MedChemExpress (MCE, USA). DSPC, cholesterol, and PEG-DSPE were purchased from Lipoid, Germany. BNP was purchased from Aladdin, China. FITC-goat antirabbit IgG, HRP-goat antirabbit IgG were purchased from Biodragon, China.

### Cell Line and Animals

Sp2/0 cells were purchased from the American Type Culture Collection. The culture medium was DMEM (Sigma, USA) containing 10% FBS (Auckland BioScience, New Zealand). Newborn rabbits were obtained from Jiangsu Academy of Agricultural Sciences (JAAS, CN).

### Preparation of HDACi Lipidosomes

SAHA-loaded liposomes were prepared using a thin-film hydration method, as previously described.^26,27^ The liposomes were composed of DSPC/cholesterol/PEG-DSPE in an 8:1:1 weight ratio. SAHA was mixed with the lipids in a weight ratio of 1:9.

A 50 mg portion of the mixture was dissolved in 5 mL of chloroform in a round-bottomed flask and incubated for 4 h with continuous stirring at 40 °C. The solvent was evaporated in vacuo to form a thin film. Then, the resulting dry film was hydrated with 5 mL of PBS (pH 7.4) along with 2% v/v tween 80 to make the lipid film completely hydrated to form a liposomal dispersion. The liposomal suspension was further centrifuged at 8000 rpm for 10 min to remove the unencapsulated SAHA using ultrafiltration centrifugation. Liposomes were washed with PBS (pH 7.4) containing 50 mg/mL trehalose as a cryoprotectant, then lyophilized, and stored in 4–8 °C. C6-loaded liposomes (C6-LP) were also prepared by using the film dispersion method mentioned above.

### Characterization of SAHA-LP and C6-LP

The particle size, polydispersity, and zeta potential of SAHA-LP and C6-LP were measured using DLS and electrophoretic light scattering techniques (Zetasizer Nano ZS90, Malvern). The morphology of SAHA-LP and C6-LP was observed using TEM (HITACHI JEOL 2010, Japan) after the samples were stained with 2% w/v phosphotungstic acid solution. The EE, LC, and cumulative release rate of SAHA were measured using a UV spectrophotometer (Nanodrop 2000, Thermo) and an HPLC system (Shimadzu, Japan) at 240 nm wavelength. The EE (%) and LC (%) were calculated using the following equations, where *C*_L_ and *C*_0_ stand for the loaded SAHA concentration and the original SAHA concentration, *W*_SAHA_ and *W*_lipo_ stand for the weight of the loaded SAHA and liposomes.^28^

1 $$EE\;\left(\%\right){=C}_{L}{/C}_{0}\times 100\%$$

2 $$LC\;\left(\%\right)=WSAHA/(W_{SAHA}+W_{lipo})\times 100\%$$

The in vitro release of SAHA-LP in PBS and PBS containing 10% FBS was investigated using the dialysis diffusion method at 37 °C over 84 h. One milliliter of SAHA-LP solution was added into each dialysis bag (MWCO 3500 Da) and placed in 10 mL of the above two kinds of buffer separately. At indicated time intervals of 12 h, 1 mL of the sample was withdrawn from the dialysis buffer for quantification, and an equivalent volume of fresh buffer was added into the system. The amounts of SAHA were determined using a UV spectrophotometer at 240 nm, and the cumulative drug release rate was calculated using the following equation:

3 $$Er\;\left(\%\right)=\left[V_{i}(C_{1}+C_{2}+\cdot \cdot \cdot +C_{i-1})+{VC}_{i}\right]/M\times 100\%$$

where *C*_*i*_ and *V*_*i*_ stand for the SAHA concentration and volume of the sample, *V* represents the volume of the dialysis buffer, *M* is the total weight of SAHA loaded in the liposomes, and Er is the cumulative release percentage.

### Liposome Internalization and Retention Study

SP2/0 cells were incubated with the fluorescently labeled liposomes (C6-LP) for different times at 37 °C. Then, the cells were washed with PBS to remove unbound liposomes.

Finally, the cell specimens were plated to coverslips, and the fluorescence of C6-LP in the cells of different incubation times was observed using a fluorescence microscope (Zeiss Axio Vert A1).

### SAHA-LP Injection and Antigen Immunization

The newborn rabbits were treated with SAHA-LP and given intraperitoneal injections every 3 days starting from the seventh day postnatal. Each injection contained 200 μg of liposomes including 15 μg of SAHA, for a total of 4 injections. In parallel, another group was treated with free SAHA solution, with daily injections of 15 μg for a total of 12 days. Subsequently, at the age of 2 months, the rabbits were immunized with an antigen of human BNP, a peptide antigen comprising only 32 amino acids. In addition, rabbits without SAHA injection were immunized with the same antigen and served as the blank group. After three immunizations, the serum immune titer was measured, and splenocytes were collected for the following analysis of the proportions of IgG-secreting plasma cells and BNP antibody-secreting plasma cells among them.

### Antibody Secretion Detection

To evaluate the enhancement effect of SAHA-LP on rabbit antibody repertoires, immunized rabbit spleen was ground to single-cell suspension, washed, and resuspended in PBS. The cells were fixed with 4% paraformaldehyde, and then the fixed cells were permeabilized with 0.5% Triton X-100 and stained with FITC-conjugated mouse antirabbit IgG antibodies for 30 min at 25 °C. Meanwhile, the IgG+ plasma cells were separated from the spleen cells using an IgG selecting kit, and the harvested cells were stained with FITC-conjugated BNP antigen for 30 min at 25 °C. The fluorescently positive cells were observed using a fluorescence cell analyzer (Countstar Rigel S2), and the positive rate was calculated.

### RT-PCR Amplification and Sequencing of Antibody-Positive Hybridoma Cells

Rabbit splenocytes were fused with mouse SP2/0 cells to form hybridomas, as described previously.^42^ After screening the hybridoma supernatants with the antigen, seven strong positive clones were selected for antibody gene amplification and sequencing.^43^ Here, we optimized the RT-PCR method by improving a kit to amplify a gene from a small number of cells, less than 50, in one step. The variable region genes of the selected cells were amplified using this one-step RT-PCR kit without an RNA extraction step. Additionally, we prepared hybridoma cells from the rabbits that had not been treated with SAHA immunomodulator, selected five positive clones, performed RT-PCR on the variable regions, and sequenced them for comparison.

According to the gene sequencing results, the DNA sequence of the variable-region fragment of the antibodies obtained in the experimental group and the blank group was aligned by DNAMAN software.

### Recombinant mAb Preparation and Characterization

Variable-region gene fragments from rabbit hybridomas’ heavy and light chains were amplified and cloned into a pcDNA3-R vector, which was constructed from pcDNA3.1 with minor modifications. The constructed plasmids were sequenced and transfected into HEK-293 cells and cultured for 6 days.^44,45^ The supernatant was collected and purified by protein A chromatography. The antibody titer was measured by indirect ELISA with BNP antigen, and the affinity was measured by a molecular interaction analyzer (Biacore X100, Cytiva).

### FICA Establishment

FICA test strips were prepared in a traditional method. Antibodies were selected by pairing the rabbit mAbs derived from this study with the mouse mAbs prepared previously. The obtained antibodies were labeled with FITC and immobilized on the T line of the FICA test strip separately.

Each FITC-labeled antibody was paired with different capture antibodies, and the performance of each antibody pair was tested when they were applied to the chromatographic test strip to detect the BNP antigen and specimens. As the sample flows along the plane of the membrane to the T-line, the sandwich complexes of antigen, detection antibody, and capture antibody formed and the fluorescence intensities of FITC on T-line were measured by an immunofluorescence quantitative analyzer (Getein 1100). After the best pair of antibodies was selected, different concentrations of specimens (from 16 to 3132 ng/mL) were added to the sample pads of different test strips in which antibody concentrations were optimized already. The linear regression equation was established for quantitative analysis. The calibration curve was constructed by plotting the signal intensity as the ordinate (*Y*) and the concentrations of BNP as the abscissa (*X*). The limit of detection of BNP was calculated by finding the concentration at which the signal-to-noise ratio was 3 (S/N = 3).

### Statistical Analysis

Statistical analyses were conducted with GraphPad Prism 8 software (GraphPad Software, Inc., San Diego, CA). Data were presented as mean ± standard error, and all experiments were independently performed at least three times. Data were analyzed by one-way ANOVA, and differences among groups were assessed using Tukey’s posttests. An unpaired Student’s *t* test or Mann–Whitney test was used when necessary.

## References

[1] Köhler G., Milstein C. (1975). Continuous cultures of fused cells secreting antibody
of predefined specificity. Nature.

[2] Smith G. P. (1985). Filamentous
fusion phage: novel expression vectors that display cloned antigens
on the virion surface. Science.

[3] McCafferty J., Griffiths A. D., Winter G., Chiswell D. J. (1990). Phage antibodies:
filamentous phage displaying antibody variable domains. Nature.

[4] Kumada Y., Tanibata R., Yamamoto K., Noguchi H., Angelini A., Horiuchi J.-i. (2023). Development and characterization of a latex turbidimetric
immunoassay using rabbit anti-CRP single-chain Fv antibodies. J. Immunol. Methods.

[5] Winters, A. ; McFadden, K. ; Bergen, J. ; Landas, J. ; Berry, K. A. ; Gonzalez, A. ; Salimi-Moosavi, H. ; Murawsky, C. M. ; Tagari, P. ; King, C. T. Rapid single B cell antibody discovery using nanopens and structured light. In MAbs; Taylor & Francis, 2019; Vol. 11, pp 1025–1035. 31185801 10.1080/19420862.2019.1624126 PMC6748590

[6] Zhou S., Zhang R., Wen Y., Zou Y., Ding D., Bian M., Cui H., Guo J. (2024). Multifunctional Lipidated
Protein Carrier with a Built-In Adjuvant as a Universal Vaccine Platform
Potently Elevates Immunogenicity of Weak Antigens. J. Med. Chem..

[7] Yu Y., Chen Y., Wang J., Fan X., He Z., Qiao S., Hou S., Zou P. (2023). A peptide derived from
the N-terminal of NS2A for the preparation of ZIKV NS2A recognition
polyclonal antibody. J. Immunol. Methods.

[8] Reynaud, C.-A. ; Weill, J.-C. Immunoglobulin Diversification by Gene Conversion. In Encyclopedia of Immunobiology; Ratcliffe, M. J. H. , Ed.; Academic Press, 2016; pp 144–147.

[9] Becker R. S., Knight K. L. (1990). Somatic diversification
of immunoglobulin heavy chain
VDJ genes: Evidence for somatic gene conversion in rabbits. Cell.

[10] Mage R. G., Sehgal D., Schiaffella E., Anderson A. O. (1999). Gene-conversion
in rabbit B-cell ontogeny and during immune responses in splenic germinal
centers. Vet. Immunol. Immunopathol..

[11] Zhai S. K., Lanning D. K. (2013). Diversification
of the primary antibody repertoire
begins during early follicle development in the rabbit appendix. Mol. Immunol..

[12] Pospisil R., Alexander C. B., Obiakor H., Sinha R. K., Mage R. G. (2006). CD5+ B
cells are preferentially expanded in rabbit appendix: The role of
CD5 in B cell development and selection. Dev.
Comp. Immunol..

[13] Wen K., Bai Y., Wei Y., Li C., Shen J., Wang Z. (2020). Influence
of Small Molecular Property on Antibody Response. J. Agric. Food Chem..

[14] Takamura N., Seo H., Ohta K. (2021). TET3 dioxygenase modulates
gene conversion at the avian
immunoglobulin variable region via demethylation of non-CpG sites
in pseudogene templates. Genes Cells.

[15] Budzynska P. M., Kylaniemi M. K., Kallonen T., Soikkeli A. I., Nera K.-P., Lassila O., Alinikula J. (2017). Bach2 regulates AID-mediated immunoglobulin
gene conversion and somatic hypermutation in DT40 B cells. Eur. J. Immunol..

[16] Slaughter M. J., Shanle E. K., Khan A., Chua K. F., Hong T., Boxer L. D., Allis C. D., Josefowicz S. Z., Garcia B. A., Rothbart S. B. (2021). HDAC inhibition results
in widespread alteration of the histone acetylation landscape and
BRD4 targeting to gene bodies. Cell Rep..

[17] Pili R., Quinn D. I., Hammers H. J., Monk P., George S., Dorff T. B., Olencki T., Shen L., Orillion A., Lamonica D. (2017). Immunomodulation
by Entinostat in Renal Cell
Carcinoma Patients Receiving High-Dose Interleukin 2: A Multicenter,
Single-Arm, Phase I/II Trial (NCI-CTEP# 7870) Entinostat and High-Dose
IL2 in Renal Cell Carcinoma. Clin. Cancer Res..

[18] Siegel D., Hussein M., Belani C., Robert F., Galanis E., Richon V. M., Garcia-Vargas J., Sanz-Rodriguez C., Rizvi S. (2009). Vorinostat in solid and hematologic
malignancies. J. Hematol. Oncol..

[19] Mei M., Chen L., Godfrey J., Song J., Egelston C., Puverel S., Budde L. E., Armenian S., Nikolaenko L., Nwangwu M. (2023). Pembrolizumab
plus vorinostat induces responses in
patients with Hodgkin lymphoma refractory to prior PD-1 blockade. Blood.

[20] Li Y.-q., Fan F., Wang Y.-r., Li L.-y., Cao Y.-j., Gu S.-m., Liu S.-s., Zhang Y., Wang J., Tie L. (2023). The novel small molecule
BH3 mimetic nobiletin synergizes with vorinostat
to induce apoptosis and autophagy in small cell lung cancer. Biochem. Pharmacol..

[21] Malarkey M., Toscano A. P., Bagheri M. H., Solomon J., Machado L. B., LoRusso P., Chen A., Folio L. R., Goncalves P. H. (2023). A pilot
study of volumetric and density tumor analysis of ACC patients treated
with vorinostat in a phase II clinical trial. Heliyon.

[22] Seo H., Hashimoto S.-i., Tsuchiya K., Lin W., Shibata T., Ohta K. (2006). An ex vivo
method for rapid generation of monoclonal antibodies (ADLib
system). Nat. Protoc..

[23] Le V. K. H., Pham T. P. D., Truong D. H. (2021). Delivery systems
for vorinostat in
cancer treatment: An updated review. J. Drug
Delivery Sci. Technol..

[24] Rani V., Venkatesan J., Prabhu A. (2022). Liposomes- A promising strategy for
drug delivery in anticancer applications. J.
Drug Delivery Sci. Technol..

[25] Alavi M., Hamidi M. (2019). Passive and active
targeting in cancer therapy by liposomes
and lipid nanoparticles. Drug Metab. Pers. Ther..

[26] La-Beck N. M., Liu X., Wood L. M. (2019). Harnessing Liposome Interactions With the Immune System
for the Next Breakthrough in Cancer Drug Delivery. Front. Pharmacol..

[27] Bachmann M. F., Jennings G. T. (2010). Vaccine delivery:
A matter of size, geometry, kinetics
and molecular patterns. Nat. Rev. Immunol..

[28] Gu Z., Wang Q., Shi Y., Huang Y., Zhang J., Zhang X., Lin G. (2018). Nanotechnology-mediated
immunochemotherapy
combined with docetaxel and PD-L1 antibody increase therapeutic effects
and decrease systemic toxicity. J. Controlled
Release.

[29] Zhou J., Zhang Q., Ma L., Zhang Y., Zhu T., Guo J., Cui Y., Zhang L. (2022). Analytical and clinical performance
of a rapid magnetic immunochromatographic assay for N-terminal pro-B-type
natriuretic peptide detection. J. Magn. Magn.
Mater..

[30] Kuramitsu M., Momose H., Uchida Y., Ishitsuka K., Kubota R., Tokunaga M., Utsunomiya A., Umekita K., Hashikura Y., Nosaka K. (2023). Performance
evaluation of Espline HTLV-I/II, a newly developed rapid immunochromatographic
antibody test for different diagnostic situations. Microbiol. Spectrum.

[31] Ediriweera M. K., Cho S. K. (2020). Targeting miRNAs by histone deacetylase
inhibitors
(HDACi): Rationalizing epigenetics-based therapies for breast cancer. Pharmacol. Ther..

[32] Perona M., Majdalani M. E., Rodríguez C., Nievas S., Carpano M., Rossini A., Longhino J. M., Cabrini R., Pisarev M. A., Juvenal G. J. (2020). Experimental studies of boron neutron capture
therapy (BNCT) using histone deacetylase inhibitor (HDACI) sodium
butyrate, as a complementary drug for the treatment of poorly differentiated
thyroid cancer (PDTC). Appl. Radiat. Isot..

[33] Paliwal S., Sharma J., Dave V., Sharma S., Verma K., Tak K., Kakarla R. R., Sadhu V., Walvekar P., Aminabhavi T. (2024). Novel biocompatible
polymer-modified liposome nanoparticles for biomedical applications. Polym. Bull..

[34] Hashemi M., Ghadyani F., Hasani S., Olyaee Y., Raei B., Khodadadi M., Ziyarani M. F., Basti F. A., Tavakolpournegari A., Matinahmadi A. (2023). Nanoliposomes for doxorubicin delivery: Reversing
drug resistance, stimuli-responsive carriers and clinical translation. J. Drug Delivery Sci. Technol..

[35] Minocha N., Kumar V. (2022). Nanostructure system:
Liposome – A bioactive carrier in drug
delivery systems. Mater. Today: Proc..

[36] Karimifard S., Rezaei N., Jamshidifar E., Moradi Falah Langeroodi S., Abdihaji M., Mansouri A., Hosseini M., Ahmadkhani N., Rahmati Z., Heydari M. (2022). pH-Responsive Chitosan-Adorned
Niosome Nanocarriers for Co-Delivery of Drugs for Breast Cancer Therapy. ACS Appl. Nano Mater..

[37] Laune M. A., Zahidi S. A., Wiemann J. T., Yu Y. (2022). Distinct Antibacterial
Activities of Nanosized Cationic Liposomes against Gram-Negative Bacteria
Correlate with Their Heterogeneous Fusion Interactions. ACS Appl. Nano Mater..

[38] Sun G., Huang Y., Cheng X., Fang L., Wang C., Zhang Y. (2025). Ammonium Sulfate Gradient-Prepared Anlotinib-Loaded Liposomes for
Potent Tumor Therapy with Decreased Side Effect. ACS Appl. Nano Mater..

[39] Ghosh S., Roy T. (2014). Nanoparticulate drug-delivery
systems: lymphatic uptake and its gastrointestinal
applications. J. Appl. Pharm. Sci..

[40] Nishioka Y., Yoshino H. (2001). Lymphatic targeting
with nanoparticulate system. Adv. Drug Delivery
Rev..

[41] Weill J. C., Weller S., Reynaud C. A. (2023). B cell
diversification in gut-associated
lymphoid tissues: From birds to humans. J. Exp.
Med..

[42] Raybould T., Takahashi M. (1988). Production of stable rabbit-mouse hybridomas that secrete
rabbit mAb of defined specificity. Science.

[43] Li Y., Liu M., Kong Y., Guo L., Yu X., Yu W., Shen J., Wen K., Wang Z. (2022). Significantly improved
detection performances of immunoassay for ractopamine in urine based
on highly urea-tolerant rabbit monoclonal antibody. Food Chem. Toxicol..

[44] Sui S., Wang H., Song J., Tai W. (2023). Development of a spermine
lipid for transient antibody expression. Bioorg.
Med. Chem..

[45] Schafer C., Young D., Singh H., Jayakrishnan R., Banerjee S., Song Y., Dobi A., Petrovics G., Srivastava S., Srivastava S. (2023). Development and characterization
of an ETV1 rabbit monoclonal antibody for the immunohistochemical
detection of ETV1 expression in cancer tissue specimens. J. Immunol. Methods.

